# The relationship between the motivation for physical activity and the level of physical activity among medical college students: based on the mediating effect of exercise self-efficacy and the moderating effect of kinesiophobia level

**DOI:** 10.3389/fpsyg.2026.1750697

**Published:** 2026-03-04

**Authors:** Jiahe Pan, Xin Cao, Fu Li, Ruiquan Wu, Zhiyi Rong, Jing Tao, Qinging Miao, Yusai Fu, Juan Xie, Xiang Zhan, Weiwei Tang

**Affiliations:** 1 School of Health Policy and Management, Nanjing Medical University, Nanjing, China; 2 School of Public Health, Nantong University, Nantong, China; 3 Academic Affairs Department of Nanjing Medical Association, Nanjing, China; 4 Department of Medical Insurance, Nantong First People’s Hospital, Nantong, China; 5 Academic Affairs Office, Kangda College of Nanjing Medical University, Lianyungang, China; 6 The Affiliated Taizhou People's Hospital of Nanjing Medical University, Taizhou, Jiangsu, China; 7 Jiangsu Provincial Institute of Health, Nanjing Medical University, Nanjing, China

**Keywords:** college students, exercise self-efficacy, kinesiophobia, physical activity level, physical activity motivation

## Abstract

**Background:**

In modern society, health issues have gained increasing attention. Physical activity, a key factor in health maintenance, is vital for college students. As future healthcare professionals, medical students’ health impacts both their academic development and future professional performance. Therefore, studying their physical activity patterns is crucial for enhancing their health.

**Methods:**

A stratified random sampling method was used to conduct a questionnaire survey among medical college students in three universities in Jiangsu Province. The questionnaire included sociodemographic information, the revised version of the Motives for Physical Activities Measure (MPAM-R), the Exercise Self-Efficacy Scale (SEE), the Kinesiophobia Causation Scale (KCS), and the short form of the International Physical Activity Questionnaire (IPAQ).

**Results:**

There was a positive correlation between college students’ physical activity motivation and physical activity level (*r* = 0.201, *p* < 0.01). Exercise self-efficacy played a partial mediating role between physical activity motivation and physical activity level, and the mediating effect accounted for 10.732% of the total effect. The kinesiophobia level could moderate the direct path of the mediation model.

**Conclusion:**

The motivation for physical activity has a significant positive predictive effect on the level of physical activity. Exercise self-efficacy plays a partial mediating role in this relationship, and this mediation is moderated by the kinesiophobia level. Therefore, improving exercise self-efficacy and reducing the kinesiophobia level are effective ways to enhance the physical activity level of college students. It is recommended to attach great importance to this and take corresponding intervention measures.

## Introduction

In contemporary society, health issues have attracted increasing and widespread attention (Suarez-Balcazar et al., 2018). As a vital factor in maintaining and promoting health, physical activity assumes significant importance for the college student population (Bull et al., 2020). Medical college students, as future practitioners in the healthcare sector, their personal health status not only bears relevance to individual academic pursuit and development (Aaltonen et al., 2021) but also exerts a potential influence on the fulfillment of their medical duties in the future (Amano et al., 2024). Therefore, a thorough exploration of the physical activity patterns among medical college students carries substantial practical significance for enhancing the health standards of this demographic.

However, the current physical activity status of college students remains far from satisfactory in practice (Radwan et al., 2019), which is constrained by multiple factors (Graupensperger et al., 2020). Specifically, physical activity motivation, as a critical internal determinant affecting college students’ participation in physical exercises, demonstrates a complex correlation with physical activity levels, and this relationship is subject to the influence of various contextual factors (Kalajas-Tilga et al., 2020). Moreover, the role of kinesiophobia has increasingly garnered academic attention (Luque-Suarez et al., 2019), as it may moderate the association between physical activity motivation and physical activity levels (Trocoli and Botelho, 2016). Concurrently, exercise self-efficacy is also postulated to serve as a mediating factor in this relational framework (Trocoli and Botelho, 2016). An in-depth exploration of the interrelationships among these constructs is of paramount importance for formulating interventions to enhance college students’ physical activity participation and promoting their comprehensive physical and mental wellbeing.

Physical activity motivation (also known as physical exercise motivation) refers to the internal psychological factors and driving forces that prompt individuals to engage in physical activities. In sports science research, it is defined as the mechanism through which individuals meet certain needs by participating in physical activities, reflecting engagement motivations (Kalajas-Tilga et al., 2020). It influences not only participation but also exercise frequency, intensity, and duration. Studies have shown that college students’ exercise motivation is multifaceted, with diverse motivations underlying the same behavior (Deci and Ryan, 2012). Based on the cognitive evaluation theory of motivation, internal exercise motivation affects mental health by shaping participation behavior (Deci and Ryan, 2000). Against the backdrop of the comprehensive promotion of the Healthy China Strategy, researching the physical activity motivation of college students is crucial for their all-round development, as well as for the long-term development of higher education and society. Although existing studies have focused on this topic, there are still gaps (Liu et al., 2023), particularly in the lack of practical guidance on transforming exercise motivation into actual behavior. Given the defect, we aimed to better explore the relationship between physical activity motivation and physical activity levels to refine theories, with the goal of developing effective intervention strategies and providing operational guidance for universities, families, and society.

Self-efficacy refers to an individual’s belief in their ability to achieve a certain level of learning or action (Klassen and Klassen, 2018). In a sports context, this means an individual’s confidence in their ability to perform physical activities at a specific level. Previous studies have shown that exercise self-efficacy plays a crucial role in motivating exercise behavior. Individuals with higher exercise self-efficacy tend to exhibit richer positive experiences and stronger exercise motivation in sports settings (Wu et al., 2022). In recent years, the relationship between physical activity motivation and exercise self-efficacy has attracted increasing attention from scholars. Relevant research has shown that there is a significant positive correlation between physical activity motivation and exercise self-efficacy (Neace et al., 2022), and physical activity motivation not only directly affects physical activity but also indirectly affects it through exercise self-efficacy (Klompstra et al., 2018). In addition, self-efficacy is an important influencing factor of kinesiophobia (Zhang et al., 2023). Effectively improving exercise self-efficacy is of positive significance for improving the fear-avoidance behavior caused by kinesiophobia and increasing physical activity (Egerton et al., 2022).

Kinesiophobia refers to patients’ fearful psychology toward functional rehabilitation exercises and daily activities due to pain (Trocoli and Botelho, 2016). Specifically, after an individual suffers painful injury, enhanced pain sensitivity triggers excessive and irrational fear of physical activity or exercise (Vlaeyen and Linton, 2000). In Vlaeyen et al. (1995) proposed a cognitive-behavioral model of fear of movement or reinjury, explaining how individuals develop irrational fear toward movements mistakenly perceived as causing further harm. Studies at home and abroad show that kinesiophobia may lead to reduced or avoided daily physical activities, thereby decreasing activity levels and adversely affecting patients’ quality of life (Trocoli and Botelho, 2016). Evidently, kinesiophobia significantly undermines individual sports participation and quality of life. Jochimsen et al. (2020) and other scholars found that kinesiophobia patients lack confidence in their ability to perform physical activities, with lower self-efficacy, which further weakens their motivation to engage in physical activities and leads to reduced physical activity levels. This situation affects patients’ quality of life and may additionally escalate social medical burdens, thereby impeding the comprehensive advancement of sports and the implementation of the Healthy China Strategy.

Considering the unclear mechanism of action among physical activity motivation, exercise self-efficacy, kinesiophobia, and physical activity level in current research, this study took medical college students as samples to explore the influence path of physical activity motivation on physical activity level, and consider the role of exercise self-efficacy and kinesiophobia level in the relationship between them. This study distinguishes itself from recent literature on college students’ physical activity motivation and engagement by virtue of population specificity and model refinement, with a direct contrast to Sheng et al.’s widely cited work on general undergraduate populations. Sheng et al. (2025) examined the relationship between exercise motivation, self-efficacy, and physical activity among 362 Chinese college students, finding that exercise self-efficacy exerted a dominant mediating effect and that gender moderated the pathway between self-efficacy and physical activity. However, their model focused on a general student population and omitted psychological constructs specific to health-related groups, such as kinesiophobia.

In contrast, the present study targets Chinese medical college students—a cohort with unique professional attributes that have been overlooked in Sheng et al.’s framework. Methodologically, while Sheng et al. emphasized the mediating role of self-efficacy and gender differences, our study advances the model by quantifying a partial mediating effect of exercise self-efficacy and incorporating kinesiophobia as a critical moderator of the direct motivation-physical activity pathway, with a statistically significant negative moderating effect that was absent in Kim et al.’s gender-stratified model. This refinement addresses Sheng et al.’s limitation of ignoring fear-related barriers, revealing that high kinesiophobia attenuates the positive predictive effect of motivation on physical activity—a mechanism uniquely relevant to medical students.

By focusing on this understudied population and integrating a fear-related moderator with precise effect size quantification, this study fills a gap in recent research that has generalized physical activity mechanisms across college students without accounting for the professional-specific psychological correlates of future healthcare providers.

## Materials and methods

### Design

This study is a cross-sectional survey based on self-administered questionnaires, with medical college students as research subjects. The research process complies with relevant medical ethics standards.

### Study population

The minimum sample size required for this study was calculated using the stratified random sampling formula. Based on relevant literature, the expected standard deviation was 2,114.8 METs (Yang and Liu, 2022), with *α* set at 0.05 (=1.96) and the allowable error specified as 70 METs. The proportion of cases in each stratum relative to the total population was ω _1_ = *ω*_2_ = *ω*_3_ = 0.33. The calculated minimum sample size was *n* ≈ 1,276. Considering a 20% invalid questionnaire rate, the total required sample size was determined to be 1,531 cases. Calculation formula: $n=\frac{\sum W_{i}^{2}S_{i}^{2}/\omega_{i}}{V+\sum W_{i}S_{i}^{2}/N}$ , where n is the required minimum sample size, 𝑁_𝑖_ is the number of units in each stratum, 𝑆_𝑖_^2^ is the variance of the *i*-th stratum, *V* is the variance for estimating the population mean, *V* = (*δ*/*u*_*α*/2_)^2^, and *W_i_* = *N_i_*/*N*.

A questionnaire survey was conducted among college students in Jiangsu Province using stratified random sampling. Survey regions were first randomly selected, and Nanjing (Southern Jiangsu), Nantong (Central Jiangsu), and Lianyungang (Northern Jiangsu) were drawn as representatives of the three regions. Next, one university was randomly selected in each city, with its enrolled students as the survey objects. Finally, questionnaires were distributed randomly via Wenjuanxing (an online survey platform), and valid questionnaires were verified based on completeness and rationality of responses. A total of 1,561 questionnaires were distributed, yielding 1,409 valid responses, with a validity rate of 90.26%. After the recovery of valid questionnaires, the data were imported into SPSS 27.0 software for logical verification and data cleaning. Missing value analysis revealed that a small number of missing entries existed in some variables, with the overall data missing rate ≤ 5%, which meets the acceptable range for statistical analysis. In accordance with the data processing norms for quantitative research, listwise deletion was adopted in this study to ensure the reliability and accuracy of the subsequent statistical analysis results.

### Sample characteristics

Participants in this study ranged in age from 15 to 38 years, with a mean age of (20.71 ± 1.918) years, including 453 males (32.15%) and 956 females (67.85%). In terms of grade distribution, freshmen (53.94%) account for the highest proportion. Myopic individuals constituted 84.88%, with non-myopic at 15.12%. Problematic daily mobile phone screen time was prevalent at 86.66%, while normal was only 0.57% and excessive 12.77%. For sleep duration (h), 386 (27.39%) had insufficient sleep (Table 1).

**Table 1:** Characteristics of subjects (*N* = 1,409).

Characteristics	Categories	*n* (%)
Gender	Male	453 (32.15)
	Female	956 (67.85)
Grade	Freshman	760 (53.94)
	Sophomore	83 (5.89)
	Junior	459 (32.58)
	Senior	107 (7.59)
Age	≤18	14 (0.99)
	19–30	1,388 (98.51)
	≥31	7 (0.50)
Vision status	Myopic	1,196 (84.88)
	Non-myopic	213 (15.12)
BMI (kg/m^2^)	Underweight (<18.5)	228 (16.18)
	Normal weight (18.5–23.9)	755 (53.59)
	Over weight (24.0–27.9)	199 (14.12)
	Obesity (≥28.0)	227 (16.11)
Daily mobile phone screen time (h)	Normal (<2)	8 (0.57)
	Excessive (2–4)	180 (12.77)
	Problematic (>4)	1,221 (86.66)
Sleep duration (h)	Insufficient (<7)	386 (27.39)
	Optimal (7–9)	1,007 (71.47)
	Excessive (>9)	16 (1.14)

### Instrument

#### Physical activity motivation

The Measurement of Physical Activity Motivation developed by Ryan et al. is a commonly used instrument for studying exercise motivation (Richard et al., 1997). The study adopted the simplified version of the Motives for Physical Activities Measure-Revised (MPAM-R) revised by Chen et al. (2013). It includes 5 dimensions (appearance, health, fun, ability, and social motivation) with 3 items per dimension, scored on a 5-point Likert scale (1 = none to 5 = very strong). Total scores range from 15 to 75, with higher scores indicating stronger motivation. In this study, the *Cronbach′ α* coefficients of the scale and each dimension were 0.859–0.972, demonstrating good internal consistency.

#### Exercise self-efficacy

The Exercise Self-Efficacy Scale (SEE), developed by Resnick and Jenkins (2000), is used to assess patients’ level of exercise self-efficacy. It comprises 9 items rated on a 10-point scale (0 = no possibility to 10 = very possible), with higher total scores indicating greater self-efficacy (Resnick and Jenkins, 2000). The scale’s *Cronbach’s α* coefficient in this study was 0.827, demonstrating high reliability.

#### Kinesiophobia

The Kinesiophobia Causes Scale (KCS), developed by Knapik et al. (2011), is designed to assess the etiologies of kinesiophobia in adults. It includes 2 subscales (11-item Physiological Factors and 9-item Psychological Factors) and uses a 5-point Likert scale. Total scores are calculated as the average of the two subscale sums, with higher scores indicating greater kinesiophobia. In this study, the *Cronbach′ α* coefficients of the scale was 0.929, and the KMO value was 0.940.

#### Physical activity

The International Physical Activity Questionnaire Short-form (IPAQ-SF) (Pols et al., 1998) was used to assess participants’ physical activity. This questionnaire has demonstrated high reliability and validity among college students (Nascimento-Ferreira et al., 2022). Comprising 7 items, the IPAQ-SF asks participants to recall and report the frequency and duration of their high-intensity, moderate-intensity, walking, and sedentary activities in the past week. According to the IPAQ manual (Ainsworth et al., 2000), total physical activity level is calculated by summing the three intensity levels. Weekly activity volume is calculated as MET value × duration (min) × frequency (MET: 8 for high-intensity, 4 for moderate-intensity, 3.3 for walking), with three activity levels classified: Physically active (≥3,000 MET-min/week), Moderately active (600 to <3,000 MET-min/week), and Insufficiently active (<600 MET-min/week). The scale’s standardized Cronbach’s *α* coefficient in this study was 0.711.

### Data analysis

Data were logically checked, described, and statistically analyzed using SPSS 27.0 software, with *p* < 0.05 defined as statistically significant. Spearman’s correlation analysis was employed to examine the correlations among kinesiophobia level, exercise self-efficacy, physical activity motivation, and physical activity level. Mediation and moderation effect analyses were conducted using the SPSS macro program PROCESS 3.2, and Models 4 and 5 were selected to test the hypotheses.

## Results

### Descriptive statistics and correlation coefficients

Normality tests were conducted on all variables, with no variables conforming to a normal distribution. Spearman’s correlation analysis was performed for the four key variables (Table 2).

**Table 2:** Correlation analysis among physical activity motivation, exercise self-efficacy, kinesiophobia, and physical activity.

Variables	Mean ± SD	Physical activity motivation	Exercise self-efficacy	Kinesiophobia	Physical activity
Physical activity motivation	47.13 ± 14.50	1			
Exercise self-efficacy	38.72 ± 19.18	0.240^a^	1		
Kinesiophobia	56.20 ± 13.58	−0.283^a^	−0.113^a^	1	
Physical activity	3,190.78 ± 2,662.07	0.201^a^	0.135^a^	−0.293^a^	1

SD, standard deviation.

^a^*p* < 0.01.

Physical activity motivation was positively correlated with exercise self-efficacy (*r* = 0.240, *p* < 0.01) and physical activity levels (*r* = 0.201, *p* < 0.01), while negatively correlated with kinesiophobia (*r* = −0.283, *p* < 0.01). Exercise self-efficacy demonstrated a positive correlation with physical activity (*r* = 0.135, *p* < 0.01) and a negative correlation with kinesiophobia (*r* = −0.113, *p* < 0.01). Kinesiophobia was negatively correlated with physical activity (*r* = −0.293, *p* < 0.01). These correlations clarify the interrelationships among the variables, supporting further moderated mediation analysis.

### Testing for moderated mediation effect

In all effect analyses, gender, grade, vision status, BMI, daily mobile phone screen time, and sleep duration were controlled as covariates. PROCESS macro Model 4 was utilized to examine whether exercise self-efficacy mediated the relationship between physical activity motivation and physical activity. After adjusting for covariates, results (Tables 3 and 4) indicated that physical activity motivation had a significant total effect on physical activity (*B* = 0.205, *p* < 0.001), with a persistent significant positive direct effect after controlling for exercise self-efficacy (*B* = 0.183, *p* < 0.001). The 95% confidence intervals for both the direct effect of physical activity motivation [0.132, 0.235] and its indirect effect via exercise self-efficacy [0.008, 0.038] excluded zero (Table 4), confirming the statistical significance of both pathways. The direct pathway accounted for 89.268% of the total effect, while the mediated pathway contributed 10.732% (total effect: *B* = 0.205).

**Table 3:** The mediating effect of exercise self-efficacy on physical activity.

Variables	*B*	SE	*t*	*p*	LLCI	ULCI	*R* ^2^
Outcome variable: exercise self-efficacy							0.102
Physical activity motivation	0.240	0.026	9.301	<0.001^b^	0.189	0.290	
Gender	−0.337	0.055	−6.076	<0.001^b^	−0.445	−0.228	
Grade	−0.046	0.024	−1.947	0.052	−0.093	0.000	
Vision status	−0.037	0.072	−0.520	0.603	−0.178	0.103	
BMI	0.000	0.000	0.237	0.813	0.000	0.000	
Daily mobile phone screen time	−0.027	0.009	−2.838	0.005^a^	−0.045	−0.008	
Sleep duration	0.022	0.029	0.766	0.444	−0.035	0.079	
Outcome variable: physical activity							0.123
Physical activity motivation	0.183	0.026	6.975	<0.001^b^	0.132	0.235	
Exercise self-efficacy	0.091	0.026	3.443	<0.001^b^	0.039	0.143	
Gender	−0.271	0.056	−4.878	<0.001^b^	−0.379	−0.162	
Grade	−0.181	0.024	−7.676	<0.001^b^	−0.228	−0.135	
Vision status	−0.214	0.071	−3.020	0.003^a^	−0.352	−0.075	
BMI	0.000	0.000	2.359	0.019	0.000	0.000	
Daily mobile phone screen time	−0.010	0.009	−1.031	0.303	−0.028	0.009	
Sleep duration	−0.008	0.029	−0.292	0.770	−0.065	0.048	

*B*, unstandardized coefficients; SE, standard error; LLCI, lower limit of the 95% confidence interval; ULCI, upper limit of the 95% confidence interval.

^a^*p* < 0.05.

^b^*p* < 0.001.

**Table 4:** Total effect, direct effect and mediating effect.

Physical activity motivation → Physical activity					
Variable relationship	Effect	SE	LLCI	ULCI	Proportion
Total effect	0.205	0.026	0.155	0.255	
Direct effect	0.183	0.026	0.132	0.235	89.268%
Indirect effect	0.022	0.008	0.008	0.038	10.732%

Subsequently, PROCESS macro Model 5 was employed to test the moderated mediation model with covariates controlled. Results (Table 5) showed that after incorporating kinesiophobia into the model, the interaction term (physical activity motivation × kinesiophobia) exerted a significant moderating effect on physical activity (*B* = −0.052, *t* = −2.645, *p* < 0.05), indicating kinesiophobia as a moderator in the relationship between physical activity motivation and physical activity.

**Table 5:** The moderated mediation model with exercise self-efficacy as a mediator and kinesiophobia as a moderator.

Variables	*B*	SE	t	*p*	LLCI	ULCI	*R* ^2^
Outcome variable: exercise self-efficacy							0.102
Physical activity motivation	0.240	0.026	9.301	<0.001^b^	0.189	0.290	
Gender	−0.337	0.055	−6.076	<0.001^b^	−0.445	−0.228	
Grade	−0.046	0.024	−1.947	0.052	−0.093	0.000	
Vision status	−0.037	0.072	−0.520	0.603	−0.178	0.103	
BMI	0.000	0.000	0.237	0.813	0.000	0.000	
Daily mobile phone screen time	−0.027	0.009	−2.838	0.005^a^	−0.045	−0.008	
Sleep duration	0.022	0.029	0.766	0.444	−0.035	0.079	
Outcome variable: physical activity							0.180
Physical activity motivation (X)	0.130	0.026	5.014	<0.001^b^	0.079	0.181	
Exercise self-efficacy (M)	0.088	0.026	3.426	<0.001^b^	0.038	0.138	
Kinesiophobia (W)	−0.233	0.026	−9.061	<0.001^b^	−0.284	−0.183	
Physical activity motivation × Kinesiophobia (X × W)	−0.052	0.020	−2.645	0.008^a^	−0.090	−0.013	
Gender	−0.191	0.055	−3.495	<0.001^b^	−0.298	−0.084	
Grade	−0.172	0.023	−7.519	<0.001^b^	−0.217	−0.127	
Vision status	−0.198	0.069	−2.882	0.004^a^	−0.332	−0.063	
BMI	0.000	0.000	2.383	0.017	0.000	0.000	
Daily mobile phone screen time	−0.002	0.009	−0.167	0.867	−0.019	0.016	
Sleep duration	−0.018	0.028	−0.632	0.528	−0.072	0.037	

B, unstandardized coefficients; SE, standard error; LLCI, lower limit of the 95% confidence interval; ULCI, upper limit of the 95% confidence interval.

^a^*p* < 0.05.

^b^*p* < 0.001.

**Figure 1:**
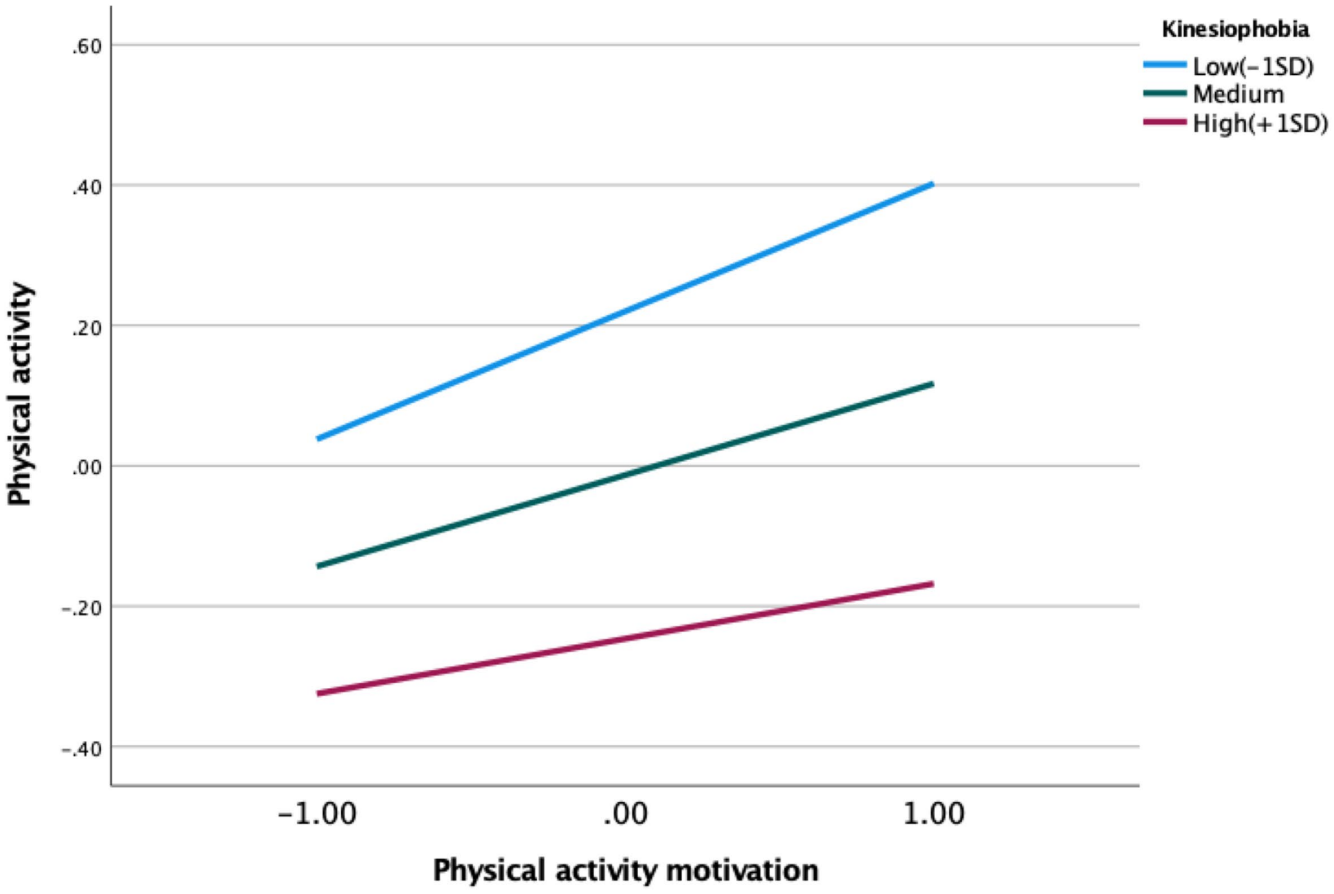
Simple slope analysis.

**Figure 2:**
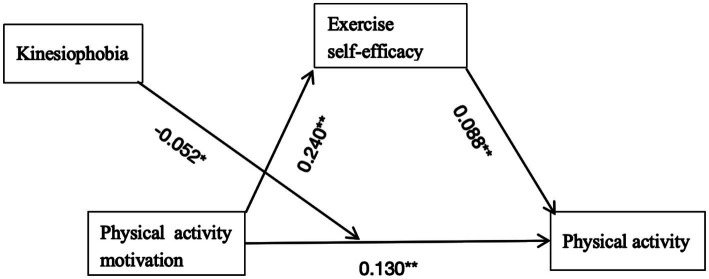
The final moderated mediation model. **p < 0.001.*p < 0.05.

Simple slope analysis (Figure 1) revealed an inverse association between kinesiophobia levels and the predictive effect of physical activity motivation on physical activity. Specific effects of kinesiophobia at different intensity levels (M-1SD, M, M + 1SD) are presented in Table 6: the positive predictive effect of physical activity motivation on physical activity was most pronounced under low kinesiophobia (*B* = 0.182, *t* = 0.032), and remained significant but weakened under high kinesiophobia (*B* = 0.078, *t* = 0.033). Rising kinesiophobia levels gradually attenuated the predictive power of physical activity motivation on physical activity. The complete moderated mediation model is illustrated in Figure 2.

**Table 6:** The mediating effect of exercise self-efficacy under different physical activity motivations.

Variable combination	Kinesiophobia	Effect	SE	LLCI	ULCI
The mediating role of exercise self-efficacy	−1 (Low)	0.182	0.032	0.119	0.246
	0 (Medium)	0.130	0.026	0.079	0.181
	1 (High)	0.078	0.033	0.014	0.143

SE, standard error; LLCI, lower limit of the 95% confidence interval; ULCI, upper limit of the 95% confidence interval.

## Discussion

This study explores the association between physical activity motivation and physical activity levels among Chinese medical college students, while examining the mediating role of exercise self-efficacy and the moderating role of kinesiophobia level. By constructing a moderated mediation model, this research clarifies the psychological correlates underlying the link between physical activity motivation and actual physical activity engagement, and provides evidence-based insights for developing targeted interventions to support physical activity levels in this specific population. The findings highlight practical implications for promoting the physical and mental wellbeing of medical college students through tailored psychosocial and behavioral strategies.

### Physical activity motivation and physical activity levels

Self-Determination Theory posits that the development of individuals’ motivation to engage in physical activity follows a continuous spectrum, progressing from amotivation to external motivation, and ultimately to internal motivation (Deci and Ryan, 2000). The findings of this study indicated that physical activity motivation positively predicts physical activity levels. Individuals with stronger physical activity motivation tend to proactively seek more exercise-related knowledge and skill training, and actively participate in various sports challenges, thereby enhancing their physical activity levels through practice. Additionally, relevant studies have shown that exercise motivation is correlated with the reinforcement and sustainability of exercise behavior (Flack et al., 2021). Individuals with high motivation are more likely to overcome difficulties and setbacks during exercise and maintain long-term participation in physical activity, with such individuals typically exhibiting higher physical activity levels. Therefore, educational institutions can start by stimulating and strengthening college students’ physical activity motivation—for instance, by promoting the benefits of exercise, offering diverse sports program options, and establishing reasonable exercise goals and reward mechanisms—to inspire students’ intrinsic motivation. Meanwhile, physical education teachers can guide students to connect physical activity with their personal needs, thereby strengthening their physical activity motivation and facilitating the transformation of exercise motivation into actual exercise behavior (Courneya et al., 2012).

### The mediating effect of exercise self-efficacy

The findings of this study demonstrate that physical activity motivation among college students is not only directly associated with their physical activity levels but also indirectly linked to them through exercise self-efficacy, with the mediating role of exercise self-efficacy accounting for 10.732% of the total predictive effect. This result aligns with Self-Efficacy Theory (Bandura, 1977), which posits that an individual’s subjective assessment of their ability to perform a specific behavior is associated with goal attainment. It also helps clarify the specific relationship between physical activity motivation and college students’ physical activity levels. From the perspective of Self-Determination Theory (Miller et al., 1988), when an individual’s needs for autonomy, competence, and relatedness are fulfilled during physical activity, they are more inclined to develop intrinsic motivation, which is associated with greater engagement in physical activity (Lewis et al., 2020). Individuals with stronger physical activity motivation—whether motivated by intrinsic interest or external goals—are more likely to actively participate in exercise (Zhao et al., 2023). In this process, with continuous attempts and progress, their confidence in their own exercise capabilities tends to strengthen; that is, their exercise self-efficacy improves (Robinson et al., 2019). As an individual’s judgment of their own exercise capabilities, exercise self-efficacy functions as a mediator: it is linked to greater enthusiasm and persistence in physical activity participation, which in turn relates to higher physical activity levels. Relevant studies have shown that the physical exercise atmosphere significantly predicts exercise self-efficacy (Zhao et al., 2023); therefore, colleges and universities should foster a positive physical exercise atmosphere, actively offer engaging and personalized emerging sports programs, and encourage college students to experience the pleasure of exercise during physical activities, thereby supporting improvements in exercise self-efficacy.

### The moderating effect of kinesiophobia

Numerous studies consistently indicate that self-efficacy and kinesiophobia levels exhibit an inverse relationship: the higher the kinesiophobia level, the lower the self-efficacy (John et al., 2023; Pina et al., 2025). According to the “Fear-Avoidance Model” (Feldberg et al., 2023), when the body experiences fear of movement due to pain, corresponding cognitive and behavioral correlates may emerge; under conditions of high kinesiophobia, the positive association between individuals’ self-efficacy and their behavior is weaker (Zhang et al., 2023). In this context, kinesiophobia levels may be directly associated with physical activity levels rather than functioning as a moderator (Balicka-Bom et al., 2021).

The findings of this study show that kinesiophobia level moderates the direct association in the mediating process through which physical activity motivation relates to physical activity levels via exercise self-efficacy. Moreover, under low kinesiophobia levels, the positive predictive effect of physical activity motivation on physical activity levels is more pronounced, indicating that high kinesiophobia levels weaken the positive association between physical activity motivation and physical activity levels. Specifically, kinesiophobia level negatively moderates the positive relationship between physical activity motivation and physical activity levels. Individuals with high physical activity motivation, under low kinesiophobia levels, are more likely to translate their motivation into actual physical activity behaviors, actively participate in various sports activities, and maintain high physical activity levels. Anxiety and depression are currently major public concerns in the context of mental health, with significant implications for population health (Guan et al., 2025; Formolo et al., 2023; Xu et al., 2025; Miao et al., 2025). Notably, individuals with high kinesiophobia levels may be more prone to negative emotions such as anxiety and depression (Pells et al., 2007), and these emotions are related to reduced motivation and confidence to engage in physical activity. Physiologically, high kinesiophobia is linked to the body’s stress responses (Ramprasad et al., 2011), such as muscle tension and increased heart rate; these physiological correlates may enhance individuals’ discomfort during exercise, which is associated with lower engagement in physical activity. Additionally, individuals with high kinesiophobia levels are more prone to associating movement with negative outcomes—when facing physical activity, their attention is more focused on potential risks (Núñez-Cortés et al., 2023) rather than the positive effects of the activity itself. This cognitive bias is associated with reduced translation of physical activity motivation into actual action (Hammer et al., 2020). In contrast, individuals with low kinesiophobia levels can view exercise more rationally and focus more on the benefits of exercise (Sarac et al., 2024); thus, driven by high physical activity motivation, they are more likely to actively engage in physical activity. Therefore, during college students’ participation in sports, providing timely positive feedback and encouragement—such as the application of mindfulness-based cognitive therapy and confidence intervention (Guetterman et al., 2015)—may alleviate negative emotions, which is associated with reduced kinesiophobia levels, strengthened physical activity motivation, and sustained maintenance of physical activity levels.

### Limitations and recommendations

This study adopted a cross-sectional design, which only reveals the associative characteristics between variables rather than inferring their causal order and direction of action. The sample has limitations in representativeness due to its single source: the participants were mainly selected from medical colleges and universities in Jiangsu Province, failing to cover college students from different geographical regions nationwide, various types of universities, and non-medical majors. The research data were mainly based on participants’ self-reports. Affected by subjective cognition, recall accuracy, and social desirability bias, there may be recall and reporting bias. Future studies could expand the geographical coverage of samples, increase the sample size, and integrate objective measurement tools to improve data objectivity, thereby enhancing the generalizability of the research conclusions.

## Conclusion

This study employed a stratified random sample of medical college students from Jiangsu Province to construct and test a moderated mediation model examining how physical activity motivation influences students’ physical activity levels. Findings revealed that physical activity motivation positively correlates with physical activity levels and exercise self-efficacy, while negatively correlating with kinesiophobia. Exercise self-efficacy played a partial mediating role in the relationship between physical activity motivation and physical activity levels (accounting for 10.732% of the total predictive effect), and kinesiophobia exerted a negative moderating effect on the direct pathway of this relationship. It is hypothesized that future intervention studies targeting medical college students’ physical activity levels could concurrently focus on strategies to enhance exercise self-efficacy and reduce kinesiophobia—such as exploring more attractive physical activity formats, designing progressive physical education courses, providing personalized exercise guidance, or organizing diverse sports competitions. This approach may facilitate the translation of physical activity motivation into sustained participation, thereby potentially improving students’ physical activity levels, which serves as a testable hypothesis for future empirical intervention research.

## Data Availability

The raw data supporting the conclusions of this article will be made available by the authors, without undue reservation.
